# Desmoplasia in Pancreatic Cancer. Can We Fight It?

**DOI:** 10.1155/2012/781765

**Published:** 2012-10-22

**Authors:** E. E. Merika, K. N. Syrigos, M. W. Saif

**Affiliations:** ^1^Oncology Unit, Third Department of Medicine, Sotiria General Hospital, University of Athens, 10679 Athens, Greece; ^2^Tufts University School of Medicine, Boston, MA 02111, USA

## Abstract

The hallmark of pancreatic tumours, the desmoplastic reaction, provides a unique microenvironment that affects pancreatic tumour behaviour, its ability to grow and metastasize as well as resist the effects of chemotherapy. Complex molecular interactions and pathways give rise to the desmoplastic reaction. Breakdown or penetration of the desmoplastic reaction may hold the key to overcoming the limits of delivery of efficacious chemotherapy or the development of new targeted treatments. Herein we discuss such new developments to fight the desmoplastic reaction, including inhibitors of the epidermal growth factor, fibroblast growth factor, the hedgehog pathway, as well as new molecular targets like CD40 agonist and its effects on T cells, extracellular matrix modifying enzymes such as LOXL2 inhibitor and novel tumour penetrating peptides for delivery of drugs.

## 1. Introduction

It is well recognised that the growth of dense, collagen-rich, extracellular matrix and stroma with high interstitial pressure around pancreatic tumours, known as the desmoplastic reaction, creates a unique microenvironment that paradoxically promotes both tumour growth and metastatic spread and at the same time forms a barrier to chemotherapy penetration. Targeting components of the tumour stroma that contribute to the desmoplastic reaction is a promising new platform of investigation. Most strategies comprise of increasingly newly identified peptides that aim to enhance chemotherapeutic and even radiotherapeutic efficacy, by increasing tumour accumulation, penetration, and drug-distribution and targeting signalling pathways, which are directly implicated in the formation of desmoplastic reaction.

The hallmark of the desmoplastic reaction in tumours originating from solid epithelial glands is a dense amount of interstitial fibrillar collagen (type I and III) and accelerated proliferation of fibroblasts. Tumour-stromal interactions between pancreatic cancer cells and stromal fibroblasts lead to enhanced key gene expression promoting primary tumour incidence, tumour growth, metastasis, and angiogenesis. The tumour cells themselves are able to produce extracellular matrix (ECM) proteins and integrins [1, 2] and interact with ECM by expressing functionally active ingredients [3, 4]. The stromal production is facilitated by an abundance of growth factors including fibroblast growth factors, epidermal growth factors receptor ligands, transforming growth factor beta isoforms, and connective tissue growth factors [5]. This environment nourishes the cancer cells and facilitates invasive and metastatic potential. In this regard, any agents that target profibrotic growth factors such as small molecule tyrosine kinase inhibitors that interfere with the epidermal growth factor (EGF) receptor, FDG, platelet-derived growth factor (PDGF) receptor signalling may be useful in suppressing the proliferation of fibroblast and stellate cells (Table 1).

## 2. Discussion

### 2.1. Transforming Growth Factor Beta (TGF*β*)

Many growth factors expressed by human pancreatic carcinoma cells have the ability to induce fibroblast proliferation, for example, transforming growth factor *β*1 (TGF*β*1) and fibroblast growth factor (FGF) 2 and are associated with advanced tumour stage and decreased survival.

TGF*β* is a potent cytokine that regulates mammalian development, differentiation, and homeostasis and normally exerts anticancer activities by prohibiting cell proliferation, motility, invasion, and metastases. In the process of tumourigenesis genetic and epigenetic events and aberrant alterations within the tumour confer TGF*β* oncogenic activities, causing direct metastatic progression via stimulation of epithelial-mesenchymal transition (EMT). EMT also confers stem cell like properties to transitioned cells such as self renewal, tumour initiating capability, and chemoresistance [6].

TGF*β* exerts its effects through TGF*β* 1 and 2 receptors (T*β*R1 and T*β*R2), and Smad transcription regulators. TGF*β* binding to T*β*R2 initiates a cascade that leads to Smad 2 and 3 activation, which in turn binds to Smad 4; the activated complex is transcriptionally active in the nucleus [7]. The growth inhibitory effect of TGF*β* is thought to be mediated by Smad-dependent TGF*β* signalling. In pancreatic defects in Smad proteins, especially Smad 4 or T*β*R2 lead to resistance to the growth inhibitory effects of TGF*β*. These events in combination with activated K-Ras result in rapid tumour development. In human pancreatic cancer cells, TGF*β*1, overexpression correlates with collagen I levels, suggesting that TGF*β*1 is directly able to elicit the desmoplastic reaction, an observation which has been confirmed in experimental models of pancreatic cancer [8]. There is also cross-talk between collagen, TGF*β*1, and MT1-MMP. MT1-MMP overexpression has been linked with fibrosis and various signalling pathways including Snail pathway, cadherins, Ras/MEK/ERK.

TGF*β* also induces Snail family of transcription factors through the Smad pathway. In PDAC, collagen activates TGF*β* signalling, in turn leading to increased Snail expression; whereas blocking TGF signalling with a highly specific T*β*RI inhibitor blocks collagen-induced Snail expression [9]. In addition, knocking down Smad 3 abrogates Snail-induced collagen fibrosis. Therefore TGF*β* is a critical signalling pathway in the development and propagation of the desmoplastic reaction. The TGF*β* pathway has been targeted using various strategies including small molecule inhibitors of T*β*RI, TGF*β*-specific neutralizing antibodies, and antisense compounds [10].

As already discussed above, TGF binding to T*β*R2 receptor leads to activation of Smad proteins which mediate gene expression related to cell growth control. Part of this effect is mediated by the Ras/MEK/ERK signalling cascade. MEK 1 inhibitor PD 98059 reduced TGF*β*1 related increase of tumour cell scattering migration and invasion [11358848] and enhances efficacy of gemcitabine. More recently, another molecule, Lefty, was identified downstream of the Ras/MEK/ERK pathway to mediate growth inhibition in pancreatic cell lines. Activation of the pathway in pancreatic cancer suppresses Lefty activation and enables cancer cells to escape growth inhibition. Inhibition of the pathway enhances TGF-mediated lefty upregulation with potential therapeutic applications [11]. The Smad pathway is also blocked by PP1 and PP2, Src family kinase inhibitors that inhibit TGF*β*-Smad signalling [12].

T*β*R1 inhibitors have also been used in combination with gemcitabine in an attempt to improve chemopenetration. Two such molecules, SB431542 and SB525334 are able to augment the cytotoxic effects of gemcitabine [13]; SB525334 also increased apoptotic cell death and affected both the AKT pathway, and T*β*R1 receptor, the former crucial in gemcitabine resistance and the latter known to affect cell migration. In a similar fashion, LY2109761 suppressed both basal and TGF*β*1-induced cell migration and invasion. In combination with gemcitabine, it reduced tumour burden, prolonged survival, and reduced spontaneous abdominal metastases [14]. The first human Phase I study of oral T*β*R1 inhibitor LY2157299 in patients with treatment-refractory malignant glioma is currently underway with promising results [15].

Another small molecule, SD-208, blocking T*β*R1, resulted in inhibition of expression of genes associated with tumour progression and inhibition of invasiveness in a cell-based assay. SD-208 treatment reduced proliferation and induced apoptosis in the primary tumours, and reduced fibrosis in the tumour microenvironment [16]. Similarly, Trabedersen (AP 12009) is a phosphorothioate antisense oligodeoxynucleotide specific for human TGF*β*2 mRNA with antitumour activity in human pancreatic cancer, such as reduction in tumour growth, lymph node metastases, and angiogenesis [17]. The T*β*R2 has also been targeted by specific neutralising antibodies. 2G8 an anti-rat monoclonal antibody specifically binds and blocks T*β*R2, inhibiting Smad 2. As a result, reducing tumour cell migration and inhibition of tumour cell migration as well as reduced EMT transcription factors are observed, which may translate in possible delayed tumour progression. This antibody has also been shown to inhibit tumour metastases in vivo [18].

More recently, further T*β*R molecular pathways have been identified such as the regulation of cell adhesive properties by decreasing expression of E cadherin. These results in increased expression of invasion associated integrins and integrin binding proteins, promoting invasion and metastasis, ECM and related protein production (collagen, fibronectin, decreases collagenase, heparinize, and stromelysins) as well as plasminogen activator inhibitor 1 and tissue inhibitor of metalloprotease that inhibit ECM degradation and increase proteolytic activity of cells [19]. Furthermore, there have been reports of significant association between plasma TGF*β*1 and overall survival in patients with locally advanced metastatic disease, Smad 4 loss correlation with lower survival with potential important implications in treatment decision [20, 21]. Clearly the increasing understanding of TGF*β* and its functions has brought a new era in molecular therapeutics. However, acquired resistance to small molecule inhibitors is a problem that has already manifested, with resultant carcinomas more aggressive and inflammatory [22]. The recent discovery that there is transcriptional talk between TGF*β* and stem cell pathways holds more promising research to come [23].

## 3. Fibroblast Growth Factor (FGF)

Another important function of TGF*β* is that it increases production of mitogenic growth factors including fibroblast growth factor. Fibroblasts are responsible for synthesis, degradation, and remodelling of ECM and can modulate behaviour of cancer cells through cytokine secretion and modification of ECM environment. Fibroblasts are thought to be mesenchymal cells, known as stellate cells, which have differentiated into myofibroblasts that secrete collagen I, which is highly resistant to proteolysis. Stellate cells are thought to mediate the invasive potential of PDAC cells and promote EMT [24] as well as resistance to radiotherapy [25]. FGF mediates its effects through different receptor isoforms. In particular, FGFR1 IIIb isoform is associated with inhibition of cancer cell proliferation, migration, and invasion, whereas FGFR1 IIIc enhances cell proliferation. FGFR2 IIIb increases venous invasion but FGFR2 IIIc is associated with metastases, more aggressive tumours and confers PDAC cells features suggestive of cancer stem cells [26]. The FGF binding protein is dramatically upregulated in pancreatic cancer and is linked to the initiation and progression of pancreatic cancer [27]. Various preclinical studies have shown FGFR signalling inhibition may play a role in inhibiting tumour growth [28]. Neutralising monoclonal antibodies to FGF2 has been shown to suppress hepatocellular cancer growth by blocking angiogenesis and inhibiting downstream cellular signalling.

## 4. CD44 and Hyaluronan

Another key role of fibroblasts in the desmoplastic reaction is hyaluronan synthesis and its interaction with CD44. CD44 is another integral cell-surface glycoprotein; overexpression of its variant forms, driven by IFN gamma, has been associated with malignant transformation of pancreatic tumours [29, 30]. In fact, pretreatment levels of CD44 and its variants have been correlated with TNM staging and may well be able to serve as tumour markers in head and neck cancers [31].

CD44 is also critical in pancreatic carcinogenesis as it is the major cell surface receptor for hyaluronan, as well as matrix metalloproteinases. Hyaluronan, is a glycosaminoglycan, able to interact with extracellular matrix molecules (hyaladherins) affecting matrix structure but also cell function through its interaction with CD44, making it another key component of the stromal reaction. In addition, its breakdown products, via hyaluronidase activity, promote angiogenesis and in turn tumour neovascularisation [32]. Hyaluronan is produced by fibroblasts in response to factors released from tumour cells, such as lactate, or by direct cell-cell contact [33]. Hyaluronan-rich stroma is associated with poor prognosis in many epithelial cancers including pancreatic and together with CD44 promotes tumour cell growth, migration, and metastases [33, 34]. It is thought that hyaluronan provides increased barrier integrity and chemoresistance through CD44-dependent reorganisation of the tumour cytoskeleton [35], where as the anti-CD44 monoclonal antibody IM7 (anti-CD44 IgG2b mAb IM7) improves vascular permeability [36]. Disruption of the hyaluronan-CD44 interaction is a key therapeutic target to prevent tumour refractoriness secondary to drug resistance [37]. One such strategy implores a hyaluronan synthesis inhibitor, 4-Methylumbelliferone (4-MU), has been shown to inhibit cell migration, proliferation, and invasion [38, 39]. The ability of 4-MU to suppress hyaluronan synthesis and accumulation has recently been linked to suppression of bone metastases in breast cancer [40]. Its inhibitory effect has been shown to slow down the development of human pancreatic cancer cell lines in vitro and in mice [41, 42] but also to enhance the efficacy of gemcitabine [43]. In a similar fashion, the action of PEGylated human recombinant PH20 hyaluronidase (PEGPH20) acting as a hyaluronan depletor improved chemopermeability of doxorubicin and gemcitabine and when given in combination with the latter led to inhibition of pancreatic tumour growth and improved survival over gemcitabine alone (median survival 28.5 days versus 15) [44, 45].

## 5. Hedgehog Pathway

**Figure 1:**
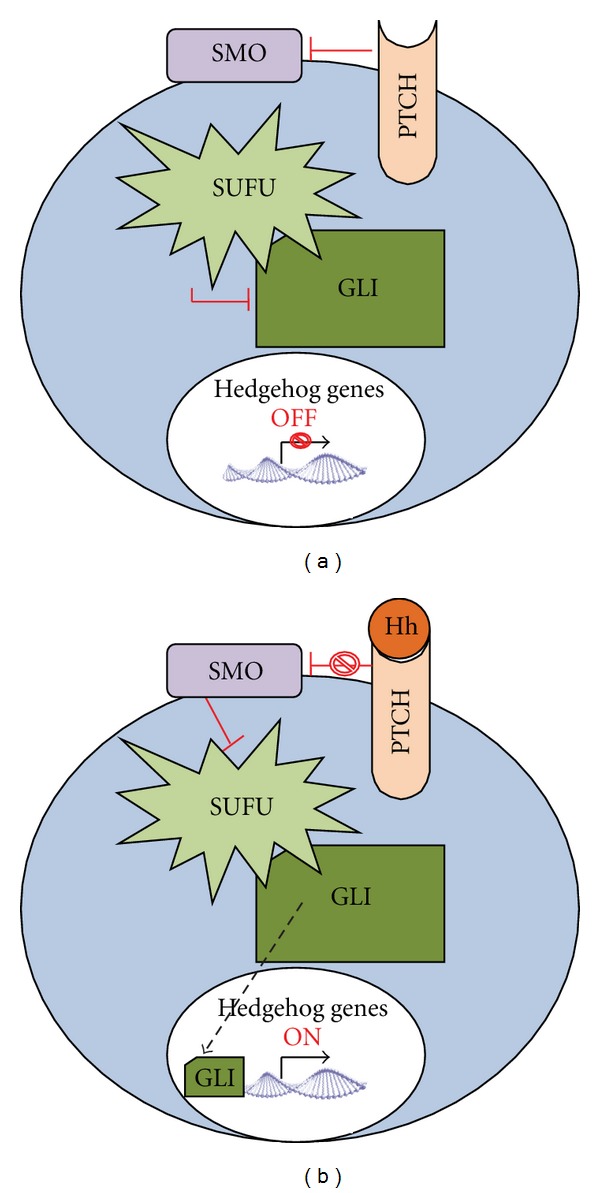
The Hedgehog pathway [46].

Hedgehog is a signalling pathway that is genetically altered and aberrantly activated in the majority of pancreatic cancers leading to tumour initiation, progression, and metastatic spread. In addition, it has been implicated in the initiation and maintenance of the desmoplastic reaction (Figure 1). The hallmark of the desmoplastic reaction is a dense amount of interstitial fibrillar collagen (type I and III) and accelerated proliferation of fibroblasts. The latter are thought to be mesenchymal cells, known as stellate cells, which have differentiated into myofibroblasts that secrete collagen I, which is highly resistant to proteolysis. Hedgehog (HH) signalling promotes myofibroblast differentiation and induces stroma-derived growth promoting molecules, which are in turn tumourigenic. In addition, HH ligands induce matrix metalloproteinases and TGF*β*1, which are both highly active in the desmoplastic reaction formation and directly involved in fibrosis. The pathway is activated when sonic hedgehog ligands (SHH) bind to the patched receptor (PTCH) relieving the inhibitory effects of Patch (PTCH) on smoothened (SMO) and activating the GL1 family of transcription factors which turn on the Hedgehog genes such as PTCH, epidermal-derived, platelet-derived, and vascular-endothelial growth factors, cyclins B, D, and E and GLI1. Bulk cancer cells secrete hedgehog ligands to activate the pathway in stroma and cancer stem cells, promoting the formation of desmoplastic reaction and facilitating maintenance of cancer stem cells involved in metastases. Ectopic production of HH ligands has been associated with pancreatic tumourigenesis [47]. In addition, overexpression of SMO in cancer-associated stromal fibroblasts has been observed that in turn activates the HH signalling pathway [48]. Evidence also suggests that tumour cells secrete HH ligand to induce tumour-promoting HH target genes in a paracrine fashion in adjacent stroma to support tumour growth [49, 50].

Blocking the hedgehog pathway in vitro studies, with the small molecule cyclopamine, a naturally occurring antagonist of the hedgehog signalling pathway component (smoothened-transmembrane receptor), leads to abrogation of pancreatic metastases and potential improvement in chemodelivery [51, 52]. IPI-926 a semisynthetic cyclopamine analogue was developed to inhibit SMO. It has been shown to reduce the desmoplastic reaction and increase tumour vascular density by blocking hedgehog signalling and hence blocking metastatic spread and tumour initiation. Inhibition of Hedgehog signalling has been shown to enhance the delivery of drugs in vitro [53] and can occur in many platforms including HH ligand inhibition, SMO antagonism, and Gli transcriptional activity inhibition.

Several studies have been designed to assess the synergistic function of Hedgehog inhibitors delivered alongside with established antineoplastic agents [54]. In one such study, Stephenson et al. tested the safety profile of IPI-926 in previously untreated metastatic pancreatic cancer in a phase Ib trial. They noted that IPI-926 facilitated the delivery of gemcitabine by diminishing tumour-associated desmoplasia with 31% of patients showing partial response and 63% showing reduction in CA 19-9. Treatment was confounded by grade 3 toxicity fatigue and transaminitis. A randomised double-blind placebo-controlled study is underway to assess survival comparison between the treatment and placebo arms, where the treatment arm will receive daily 160 mg oral IPI-926 plus gemcitabine infusion at 100 mg/m^2^ once weekly for 3 weeks of a 28-day cycle [NCT01130142]. Unfortunately the Phase II trial by Infinity was recently stopped because of futility of treatment [55].

Another SMO inhibitor, GDC-0449/Erivedge, also known as vismodegib, is an orally administrable molecule 2-arylpyridine class that inhibits SMO and is highly selective for SHH-Gli signalling, though to act by inhibiting SHH pathway at the level of Gli genes. Gli signalling has been implicated in the regulation of cell proliferation, cell cycle, and cell survival. GDC-0449 has been shown to inhibit pancreatic cancer cell viability, Gli-DNA binding and transcriptional activity and induces apoptosis in three pancreatic cancer cell lines and stem cells [56]. It also inhibited expression of HH receptors, such as Patched and SMO and effectors. Preclinical studies have demonstrated antitumour activity in xenograft models of pancreatic cancer [57]. LoRusso et al. presented their Phase I trial results in 2011 utilising GDC-0449 in patients with refractory, locally advanced or metastatic solid tumours, including 8 with pancreatic cancer [58] [21300762]. The molecule was able to produce tumour responses in 20 patients with BCC and medulloblastoma. The best observed response for pancreatic cancer was seen in one patient with stable disease at 2.8 months. Most promising was that Gli1 downregulation was noted and the treatment was associated with low toxicity. Recently following Phase II trials in BCC, the drug was approved by the FDA for the treatment of metastatic or locally advanced BCC that cannot be treated with surgery or radiotherapy. The trial showed partial response in 30% of patients with metastatic disease and complete or partial response in 43% of patients with locally advanced disease (ERIVANCE trial BCC/SHH4476g AACR). The theory behind GDC-0449 altering HH signalling is being tested in a Phase II study with vismodegib in the preoperative setting for patients with local, resectable disease to detect change in HH signalling in the normal tumour surrounding tissue (Proof of Mechanism Study of an Oral Hedgehog Inhibitor GDC-0449 in Patients With Resectable Pancreatic Ductal Adenocarcinoma in the Pre-operative Window Period, also known as HIPPoS by Cambridge University Hospitals NHS Foundation, NCT01096732, estimated primary completion date September 2012) looking at whether blocking the HH pathway will directly affect tumour cells or the surrounding normal tissue.

One of the main reasons for ultimate resistance to therapy is due to the existence of cancer stem cells which are resistant to chemotherapy and lead to treatment failure. The Michigan group are currently evaluating the combination of vismodegib with gemcitabine for patients with advanced disease and its effect to cancer stem cells and HH pathway (cancer stem cells and inhibition of HH pathway signalling in advanced pancreas cancer: a pilot study of GDC in combination with gemcitabine-NCT01195415), in a hope that pretreatment with GDC-0449 will inhibit the HH pathway in cancer cells and downstream tumour microenvironment enhancing treatment efficacy for gemcitabine. One of the primary endpoints is to evaluate the effect of HH signalling inhibition on pancreatic cancer stem cells by assessing the number of cancer stem cells before and after GDC-0449 treatment. Preliminary results of this trial show that three out of five patients who received pretreatment with GDC-0449 followed by gemcitabine treatment showed partial response, reduction in CA 19-9 levels, and increased vacuolated structures in tumour cells of one patient. The estimated primary completion date for this study is June 2013.

With a similar target in mind, another open label, single arm, multicentre Phase II trial is currently evaluating the progression free survival in patients with metastatic adenocarcinoma treated with vismodegib in combination with gemcitabine and nab-Paclitaxel (a Phase II Study of Gemcitabine and Nab-Paclitaxel in Combination With GDC-0449 (Hedgehog Inhibitor) in Patients With Previously Untreated Metastatic Adenocarcinoma of the Pancreas by Sidney Kimmel comprehensive Cancer Centre at John Hopkins-NCT01088815, estimated primary completion date December 2012). Abraxane is thought to weaken the stroma allowing for better chemotherapeutic efficacy of gemcitabine, using GDC-0449 to destroy the stroma but also to kill cancer stem cells. Furthermore Abraxane has shown clinical activity in patients overexpressing secreted protein acidic and rich in cysteine (SPARC), as it binds to the albumin portion of paclitaxel, potentially providing a tool to reverse gemcitabine resistance. Measurement of SPARC levels may also serve as a prognostic factor for treatment success [59, 60].

Other Phase I trials currently underway are assessing combination treatments with GDC-0449 such as in combination with Sirolimus or Erlotinib and Gemcitabine. Preliminary results are encouraging and have shown disease stabilisation and low drug-related toxicities (DLTs) for Erlotinib with Gemcitabine and GDC-0449. (Gemcitabine Hydrochloride With or Without GDC-0449 in Treating Patients With Recurrent or Metastatic Pancreatic Cancer by University of Chicago NCT01064622 to assess progression free survival; Sirolimus and Vismodegib in Treating Patients With Solid Tumours or Pancreatic Cancer That is Metastatic or Cannot Be Removed By Surgery by Mayo Clinic NCT01537107, primary completion date January 2014; GDC-0449 and Erlotinib Hydrochloride With or Without Gemcitabine Hydrochloride in Treating Patients With Metastatic Pancreatic Cancer or Solid Tumours That Cannot Be Removed by Surgery by Mayo clinic NCT00878163). Preliminary results are showing stable disease and low DLTs [61].

An important consideration is that SMO is localised in the primary cilium of the cell, which is critical in HH signalling and cancer progression. Primary cilia are required for the activation of the HH pathway in normal cells but are lost in many cancers. Some drugs may be ineffective in the absence of primary cilia [62]. Hence further research into overcoming this barrier should be considered when designing new platforms.

## 6. iRGD: a Tumour Penetrating Peptide for Peptide-Mediated Delivery of Drugs

One of the main reasons for treatment failure remains inability to penetrate the stromal reaction and the generation of elevated intratumour interstitial pressure. Crossing the vascular wall and penetrating into the tumour parenchyma is the main challenge for efficacious drug delivery. Recent attention has been paid to penetrating peptides for peptide-mediated drug delivery, especially peptides containing an RGD integrin recognition motif which allows them to bind to av integrins on the tumour cell surface. However to date, conventional RGD peptides have only been able to penetrate blood vessels but not the extravascular tumour parenchyma. A newly devised peptide, iRGD, a disulfide-based cyclic RGD peptide, seems to have overcome this obstacle by also targeting a downstream receptor, neuropilin-1. iRGD is a synthetic peptide containing a motif that binds to av integrins on tumour endothelium. Upon binding, the peptide is proteolytically cleaved to expose a CRGDK fragment, losing its integrin affinity but gaining affinity for neuropilin-1 instead. The new complex triggers tissue penetration, thus this peptide penetrates through the tumour vasculature into the tumour parenchyma [63].

Since the peptide is able to penetrate into the tumour parenchyma, coupling of the peptide with drugs may improve the drug delivery and efficacy, especially as iRGD seems to home to tumours but not normal tissue. av integrin and neuropilin-1 expression is largely restricted to tumours but most importantly the response is tumour specific because the peptide cleavage will only occur if there has been prior integrin activation. The hypothesis has been tested in mouse tumour models including pancreatic adenocarcinoma where various drugs including doxorubicin, nab-paclitaxel (abraxane), and doxorubicin liposomes as well as trastuzumab were coadministered with the peptide, without the need for chemical conjugation therefore preserving drug activity and improving tolerability. Tumour accumulation was increased 12-fold for abraxane, 14-fold for the doxorubicin liposomal nanoparticle, and 7-fold for the free drug and 40-fold for trastuzumab indicating that iRGD leads to enhance drug delivery to cancer cells [64]. The manufacturing company has already initiated SBIR trials with iRGD in combination with gemcitabine with preliminary data showing that iRGD enhances the anti-tumoural activity of gemcitabine in orthotopic models of pancreatic cancer [65].

## 7. CD40 Agonist

CD40 is a type I transmembrane glycoprotein receptor of the TNF-receptor superfamily widely expressed by immune cells such as dendritic cells, B cells, and macrophages but also endothelial cells, smooth muscle cells, fibroblasts, and epithelial cells. The CD40 ligand (CD40L) primarily expressed in the surface of activated T cells interacts with CD40+ B cells to produce multiple regulatory signals including T-cell and B-cell-dependent proliferation, immunoglobulin production and switching, and apoptosis. CD40L+T cells augment the antigen-presenting function of CD40+ B cells and other antigen-presenting cells (APCs) generating a number of interactions between CD4 and CD8 T cells [66, 67].

Interestingly, CD40 is also expressed in the membrane and cytoplasm of tumour cells but is absent from non-proliferating tissues. Its activation promotes apoptotic death and generation of tumour specific T-cell responses that contribute to tumour elimination [68]. The exact mechanism of CD40-CD40L interaction is still unclear as CD40 expression has been correlated with worse tumour prognosis, TNM stage, and lymph node metastases, perhaps because the CD40L is rarely expressed on pancreatic cancer TILs and hence unable to downregulate CD40+ cancer growth. In fact, presence of CD40L expression has been linked to improved survival [69]. In addition, epigenetic alterations of miRNA-regulated CD40 expression lead to downregulation of CD40 expression in pancreatic cancer cells promoting invasion and metastasis [70]. CD40 also engages in endothelial cells to induce in vitro tubule formation and expression of matrix metalloproteinases [71]. In a recent Phase I trial by He et al. [72], recombinant soluble human CD40L was used to block CD40 and demonstrated significant growth inhibitory effect in vitro. Specifically they showed the ligand was able to cause not only growth arrest but also cancer cell apoptosis. CD40 binding antibodies have the potential to modulate pancreatic cancer cell growth. Binding of recombinant soluble CD40L or with a CD40 reactive monoclonal antibody may produce a direct inhibitory effect on cancer cells. CD40 agonist antibody CP-870,893 can achieve substantial regression of tumours in some patients with inoperable pancreatic binding antibodies may bind to epitopes distinct from those involved in the natural CD40-CD40L interaction. Similarly CD40 monoclonal antibodies may cause collateral activation of antibody dependent cellular cytotoxicity.

CP-870,893 is a fully human IgG2 antibody that selectively interacts with CD40 at a distinct site from its ligand-binding region. Binding enhances MHCII expression as well as dendritic cell activity and is therapeutically effective against several CD40 + human tumours. In a Phase 1 dose escalation open label study CP-870,893 was combined with gemcitabine in patients with chemotherapy naive surgically incurable pancreatic cancer [73], tumour regression was observed a subsequent mouse model that tumour regression was T cell and gemcitabine independent but dependent on macrophages, that infiltrated the tumour and facilitated the depletion of the tumour stroma. Soon underway a small open label single-arm Phase I study looking at preoperative gemcitabine together with CP870,893 followed by addition of CP-870,893 to adjuvant chemoradiotherapy for patients with newly diagnosed resectable pancreatic cancer. Patients will receive standard surgery followed by chemoradiotherapy; one dose of gemcitabine/CP870,893 will be preoperatively and 3 doses postoperatively.

## 8. LOX-L2

Lysyl oxidase like 2 belongs to the lysyl oxidase family of extracellular matrix modifying enzymes. This group of enzymes plays an important role in connective tissue biogenesis, cellular adhesion, motility and migration, gene transcription regulation, and senescence, as well as cancer progression. Increased LOX-L2 expression has been identified in many cancers including the pancreas. In breast cancer, high levels of LOX-L2 expression appear to correlate with decreased overall survival and metastases free survival (*P* = 0.023 and *P* = 0.0367, resp.) [74]. Interestingly, LOX-L2 does not appear to be required for primary tumour growth but enables metastases in vivo.

LOXL2 serves as an extracellular matrix metalloenzyme and has been shown to catalyse the first step in the formation of crosslinks in fibrillar collagen and elastin [75, 76]. Cross-linking of collagen activates other enzymes involved in matrix remodelling such as MMPs, enhancing tumour cell invasion [77]. Therefore LOX-L2 is directly able to modify the ECM, and its overexpression leads to propagation of the desmoplastic reaction. Positive association between LOX-L2, TIMP1, and MMP9 has also been noted in human colorectal cancer [78–80]. LOXL2 inhibition has also been associated with reduction in activated fibroblasts, endothelial cells, desmoplasia, and decrease in transforming growth factor-beta signalling making LOX-L2 a potential target for fighting the desmoplastic reaction [81].

Preclinical evidence suggests that in vivo blocking LOXL2 both in vivo and in vitro is highly effective in preventing distant metastases in breast cancer through regulation of tissue inhibitor of metalloproteinase 1 (TIMP1), leading to increased TIMP1 and MMP 9 activity and facilitating ECM remodelling [82].

In pancreatic cancer cell lines, gene silencing by inhibition with small interfering RNAs has been shown to result not only in cell death but also in increased sensitivity to gemcitabine treatment [83]. In this study, LOXL2 appeared to regulate E2F5 transcription factor associated with invasion and metastases. Blocking not only LOXL2 but its effectors too, such as E2F5 or even RAMP3, a molecule downstream of LOXL2 thought to mediate some of its tumourigenic activity [81], might also prove beneficial as antitumourigenic agents.

In addition, development of specific allosteric inhibitors of LOXL2, such as AB0023, bind remote to its catalytic domain, allowing inhibition of LOXL2 regardless of substrate concentration [84]. This concept has many prospects: the ability to confer a molecule high specificity and selectivity for the cancer without affecting normal tissues, development of high affinity binders, and using different specificities of LOXL2 targeting antibodies to alter the outcome.

More excitingly, recently an intracellular function of LOXL2 has been described for the first time in relation to E-cadherin and histone H3; In normal cells, methylation of lysine 4 within histone 3 activates CDH1 transcription and E-cadherin formation, while histone deacetylation plays an important role in downregulation of E-cadherin in human pancreatic cancer promoting tumour cell migration and proliferation [85]. Loss of the cell adhesion molecule E-cadherin is critical in pancreatic tumourigenesis. LOXL2 has been found to act in the nucleus of cancer cells and deaminates the lysine 4 amino group of H3 leading to downregulation of CDH1, decreased E-cadherin expression, fewer cellular adhesions facilitating tumour growth and metastases [86].

## 9. Radiotherapy

As already mentioned above, there is data suggesting that pancreatic stellate cells confer protection against radiotherapy through *β*1-integrin and FAK signaling [25]. *β*1-integrin signaling and in particular integrin-mediated adhesion to extracellular matrix proteins has been implicated in mediating cell survival in response to radiation in different cancer cell lines [87]. Other PSC-specific matrix proteins such as periostin, stimulate growth, and confer resistance even under the effects of radiotherapy, continuing to enhance the desmoplastic reaction by producing excessive extracellyular matrix proteins [88]. Inhibition of the pathway enhances the efficacy of radiotherapy [30, 89]. More recently the role of caveolin-1 (Cav-1) as a critical signaling molecule within the *β*1-integrin and FAK pathway was described. Knockdown models of caveolin-1 increased radiosensitisation in human pancreatic cell lines [90]. Further research in this domain is required to enhance in vivo radiosensitivity.

## 10. Conclusion

Increasing understanding of the desmoplastic reaction and the heterogeneity of alterations of signalling pathways in pancreatic cancer is already providing us with new insights into how to fight desmoplasia. Preliminary evidence encourages the idea that attenuating the desmoplastic reaction may help limit the molecular and clinical course of pancreatic cancer, contain its progression, and enhance the response to chemotherapy. There is a long way to go until this evidence will become practice.

## Figures and Tables

**Table 1:** Classification of antidesmoplastic agents.

Agent	Class
PD 98059	MEK 1 inhibitor
U0126	MEK inhibitor
LY294002	ERK inhibitor
PP1-PP2	T*β*R inhibitors
SB431542 and SB525334	T*β*RI selective inhibitor
LY2109761	T*β*RI/II dual inhibitor
SD-208	T*β*RI inhibitor
AP 12009	TGF*β*2 mRNA phosphorothioate antisense oligodeoxynucleotide
2G8	Neutralising antibody to T*β*R2 neutralising antibody
IPI-926	SMO Semisynthetic cyclopamine analogue inhibitor
GDC-0449	2-arylpyridine class SMO inhibitor
iRGD	Disulfide-based cyclic RGD tumour-penetrating peptide
CP870,893	IgG2 antibody to CD40
AB0023	Allosteric inhibitor of LOX-L2
